# Second Language Use Facilitates Implicit Emotion Regulation via Content Labeling

**DOI:** 10.3389/fpsyg.2017.00366

**Published:** 2017-03-16

**Authors:** Carmen Morawetz, Yulia Oganian, Ulrike Schlickeiser, Arthur M. Jacobs, Hauke R. Heekeren

**Affiliations:** ^1^Department of Education and Psychology, Freie Universität Berlin, BerlinGermany; ^2^Center for Cognitive Neuroscience Berlin, Freie Universität Berlin, BerlinGermany; ^3^Bernstein Center for Computational Neuroscience Berlin, BerlinGermany

**Keywords:** emotion regulation, reappraisal, content labeling, L2 advantage, emotional distance

## Abstract

Previous studies reported that negative stimuli induced less affect in bilinguals when stimuli were presented in bilinguals’ second, weaker language (L2) than when they were presented in their native language (L1). This effect of L2 use was attributed to increased emotional distance as well as to increased levels of cognitive control during L2 use. Here we investigated how explicit (cognitive reappraisal, i.e., reinterpreting the meaning of the emotional stimulus to alter its emotional impact) and implicit (content labeling, i.e., categorizing the content of the image; and emotion labeling, i.e., naming the emotion induced by the emotional stimulus) emotion regulation strategies are altered in an L2 (English) context in German native speakers with medium to high proficiency in their L2. While previous studies used linguistic stimuli, such as words, to induce affect, here we used images to test whether reduced affect could also be observed for non-linguistic stimuli when presented in an L2 context. We hypothesized that the previously implicated increase in emotional distance and cognitive control in an L2 would result in an *L2 advantage* in emotion regulation (i.e., leading to less negative emotions compared to an L1 context), by strengthening the effect of linguistic re-evaluation on the evoked emotions. Using a classic emotion regulation paradigm, we examined changes in subjective emotional state ratings during reappraisal, emotion labeling and content labeling in a L1 and L2 context. We found that the strength of evoked affective responses did not depend on the language context in which an image was presented. Crucially, content labeling in L2 was more effective than in L1, whereas emotion labeling did not differ between languages. Overall, evoked responses were regulated most effectively through explicit emotion regulation (reappraisal) in L1 and L2 context. These results demonstrate an L2 advantage effect for emotion regulation through content labeling and suggest that L2 context alters sub-processes implicated in content labeling but not emotion labeling.

## Introduction

As more and more humans use a second language (L2) routinely to negotiate their everyday lives, the effects of L2 use on affective and cognitive processing have moved into the focus of intensive research (Pavlenko, 2012; Costa and Sebastián-Gallés, 2014). Previous research explored emotional word processing in an L2 (Caldwell-Harris, 2015) as well as the effects of L2 use on other cognitive domains such as decision making under risk and morality judgments (Hayakawa et al., 2016). In the present study we aim to extend this research by investigating the effect of L2 use on emotion regulation, a process at the interface of cognitive and affective processing.

Behavioral findings on second language effects on affective processing have been mixed, fueling the debate about their existence. For example, many studies of affect ratings in L1 and L2 found reduced skin conductance responses (SCR) for emotional L2 expressions, but no differences in ratings (Caldwell-Harris et al., 2011). A notable exception is a study by Hsu et al. (2015) who employed whole emotional paragraphs and found attenuated affect ratings as well as differences in brain activations. Another example is the emotional stroop task, where behavioral interference effects do not differ between languages, but SCR is reduced for L2 stimuli (Eilola and Havelka, 2011; Grabovac and Pléh, 2014). The effects of foreign language use have also been discussed in relation to embodied cognition, a theory that posits reactivation of sensory-motor traces during semantic, and in particular emotional processing (Pulvermüller, 2005; Winkielman et al., 2008; Pulvermüller and Fadiga, 2010). It was found that motor activation for neural and positive stimulation was unaffected by foreign language, the effect of negative emotion sentences on facial musculature was reduced in L2 as compared to L1 use (in a vertical stroop task: Dudschig et al., 2014, and for passive exposure to emotional sentences: Foroni, 2015). This finding aligns with other recent reports of reduced impact of negative information when presented in a foreign language (Wu and Thierry, 2013; Jończyk et al., 2016).

Furthermore, second language effects have also been reported in the other domains. While some studies found that L2 use altered risky decision making and morality judgments toward a more rational approach (Keysar et al., 2012; Gao et al., 2015; Geipel et al., 2015), other found that language switching was necessary to alter behavior (Gao et al., 2015; Oganian et al., 2016).

These mixed and diverse findings can be explained by two different mechanisms. First, at the linguistic level, it was suggested that the link between lexico-semantic representations and affect is weaker in the L2 than in the native language, due to lower subjective word frequencies and less frequent use of emotional words in the L2 (Opitz and Degner, 2012). The second approach suggests that processing of information in the L2 is less automatic, increasing cognitive load and reducing emotionality (Pavlenko, 2012). At the same time the need to inhibit native language representations leads to enhanced cognitive control (Gao et al., 2015; Jończyk et al., 2016). While the first approach suggests that second language effects should be confined to the processing of verbal material, the latter predicts effects of second language not only during linguistic tasks, but also for other cognitive tasks in second language settings, in line with recent findings (Hayakawa et al., 2016; Oganian et al., 2016). This suggests that the cognitive control of emotional responses, namely emotion regulation, might also be enhanced during L2 use, a possibility that has not yet been addressed.

During emotion regulation, the occurrence, intensity, and duration of affect is monitored, evaluated and modified (Thompson, 1994), typically resulting in down-regulation of strong emotions. Contemporary dual-process models of emotion regulation contrast deliberate/explicit (also called effortful, conscious or controlled) emotion regulation processes with automatic and implicit (also called incidental or unconscious) emotion regulation processes (Koole, 2009; Gyurak et al., 2011). However, implicit emotion regulation is not automatic *per se*, i.e., without any voluntary intention. It is important to note, that emotion regulation goals can also be implicitly activated (Williams et al., 2009; Tamir et al., 2013; Koole et al., 2015). Explicit and implicit emotion regulation are tightly linked to language processes as both could be thought of as intrinsically linguistic, i.e., mediated by inner speech (Vygotsky, 1962; Zivin, 1979; Diaz and Berk, 1992; De Guerrero, 2005; Zelazo and Cunningham, 2007): They likely involve verbal and semantic processing associated with self-related inner speech (Ochsner and Gross, 2005; Berkman and Lieberman, 2009; Kalisch, 2009; Viviani et al., 2010; Benelli et al., 2012; Burklund et al., 2014; Kohn et al., 2014; Kross et al., 2014; Messina et al., 2015; Morawetz et al., 2016). The involvement of linguistic processing in emotion regulation provides a putative link to second language use. If additional involvement of control processes increases the subjective distance to affective stimuli during L2 use, then emotion regulation in a second language should be more effective. The effectiveness of emotion regulation in a second language setting has not yet been addressed experimentally, despite the importance of this topic for psychotherapeutic approaches (Griner and Smith, 2006).

The most prominent method of explicit emotion regulation is cognitive reappraisal, by means of, covert or overt, description (Ochsner and Gross, 2008). It includes the cognitive re-evaluation of the meaning, cause, consequence, or personal significance of an emotionally arousing event in order to alter its emotional impact (Gross, 2002). Implicit emotion regulation can be achieved as a by-product of certain intentional tasks, without individuals’ awareness of the modulatory effect of the task on their emotional state (Gyurak et al., 2011). For example, labeling the non-emotional content of an emotional stimulus (content labeling), reduce the strength of elicited affect for positive and negative pictures (Constantinou et al., 2015). This strategy has also been described as distraction because the focus of attention is shifted away from the emotional stimulus to different non-emotional aspects. This implies that distraction represents an active process in which participants effortfully divert attention from the emotional stimulus. However, content labeling can be considered a more passive way of distraction as participants are not explicitly told to use content labeling to distract themselves but rather are instructed to engage with a task that is unrelated to the emotional stimulus. Not only the focus of attention could help to implicitly regulate emotions, but also emotion labeling could be an effective way to manage unwanted emotions and decrease emotional reactivity. In contrast to reappraisal, labeling emotions, i.e., putting feelings into words, relates specifically to the emotional aspect of the situation and involves an explicit verbal process of identifying and naming the emotion (Lieberman et al., 2007). Emotion labeling might be similar to distraction, as is allows to deal with highly intense emotional situations (Tabibnia et al., 2008; Kircanski et al., 2012), but unlike distraction, it also includes a learning aspect, since it requires an individual to attend to the emotional stimulus (Moyal et al., 2014).

Here, we aimed to shed light on emotion regulatory processes in a second language context. To this end we examined self-reported emotional responses to highly aversive pictures under conditions of explicit regulation through reappraisal (Morawetz et al., 2016) and implicit emotion regulation through emotion and content labeling (Constantinou et al., 2015), whereby emotion regulation was performed in either a native (German) or second (English) language context. Notably, previous studies of affective processing in a second language context used the term ‘second language context’ to describe a situation in which relevant stimuli are presented in a foreign language (e.g., Hsu et al., 2015), such that, to date, studies of affective processing in a second language context employed linguistic stimuli in the foreign language to induce affect. However, we are frequently confronted with non-linguistic affective inputs, such as images, embedded in linguistic context. Our design also allows elucidating the effects of non-emotional second language context on the processing of non-linguistic emotional stimuli.

Based on previous studies, we expected that reappraisal would reduce emotions most effectively (Lieberman et al., 2011; Burklund et al., 2014) and that content labeling would be more effective than emotion labeling (Constantinou et al., 2015). Moreover, we were interested to see whether the magnitude of evoked affective responses would differ between language contexts. Our central research questions, however, pertain to the difference between emotion regulation in L1 and L2. We hypothesized that the previously implicated weaker link between lexico-semantic representations and affect in an L2 would facilitate regulation efforts, by strengthening the effect of linguistic re-evaluation on the evoked emotions. Additionally, increased involvement of cognitive control during L2 use could further facilitate emotion regulation. In other words, the L2 context could result in increased distraction from the actual emotional stimulus thereby making emotion regulation easier. We thus expected that these factors would result in an *L2 advantage* in emotion regulation. However, we expected that the *L2 advantage* would be smallest during reappraisal, as inner speech is likely naturally triggered in L1. We further expected an *L2 advantage* effect in both labeling conditions, i.e., less negative emotional state ratings in the second compared to the native language context.

## Methods and Materials

### Participants

Thirty-seven (25 female, mean age = 25.9 years *SD* = 6.7) native speakers of German (L1) with medium to high proficiency in their second language English (L2) gave written, informed consent and participated in the study. Participants were recruited through advertisement on campus and were students at the Freie Universitaet Berlin. All participants had studied English as their first foreign language in high school (as ensured with an online questionnaire prior to the experiment, see below). Participants had normal or corrected to normal vision. The study was approved by the local ethics committee of the Psychology Department of Freie Universitaet Berlin. As we were interested in the effect of second language on emotion regulation, we only included participants who demonstrated the ability to regulate emotions and reported negative emotions when looking at highly aversive images in their native language. We only included the L1 condition in the exclusion criteria to ensure that exclusion would not be due to L2 effects.

Thus, for analysis of the foreign language effect on emotion regulation, twelve participants with low emotion regulation ability (exclusion criterion 1) and three participants with decreased emotional responses (exclusion criterion 2) were excluded [exclusion criterion: (1) mean ratings during *Decrease* condition in L1 < mean ratings during *Maintain* condition in L1, i.e., (Maintain – Decrease <0); (2) mean ratings during the *Maintain* condition >0]. The final analyses included the remaining 22 participants (14 female, mean age = 26.36 years *SD* = 7.50).

### Assessment of Language Skills

All participants completed an online language history questionnaire prior to participation (adapted from Li et al., 2006), with self-reports of L2 proficiency on a 1–7 Likert scale (1 = “single words,” 7 = ”native-like”), separately for reading, writing, speaking and listening abilities. In addition, participants’ general proficiency was also assessed after the experiment using the LEXTALE tests of German and English (Lemhöfer and Broersma, 2012). The tests consist of short lexical decision tasks, which include words of varying frequency and pseudo-words. The final score is the average percentage of correct responses to words and pseudo-words. Participants’ language profiles are summarized in **Table 1**.

**Table 1:** Participants’ language proficiency.

	Self-reported proficiency	
L2	Mean	*SD*
Overall English	5.18	0.90
Reading	5.77	0.68
Writing	4.90	0.92
Speaking	4.90	1.01
Listening	5.50	0.67

	**Lextale**	

German	91.74	5.98
English	79.05	13.08

### Personality Questionnaires

Participants rated their state anxiety before and after the experiment using the STAI (Spielberger et al., 1970; Laux et al., 1981) in order to assess any changes in their emotional state. The STAI is a self-report scale for measuring two distinct anxiety concepts: state anxiety and trait anxiety. Each scale comprises 20 statements regarding anxiety. State anxiety items include, e.g., “I am tense”; “I am worried” and “I feel calm”; “I feel secure.” Personality characteristics were measured using the NEO-Five Factory Inventory (Borkenau and Ostendorf, 1999). The NEO-FFI assesses the five-factor model of personality (Big Five), that assumes that personality can be described along five dimensions (Costa and MacCrae, 1992; McCrae and John, 1992; McCrae and Costa, 2004): (1) *neuroticism* (e.g., “I have frequent mood swings”); (2) *extraversion* (e.g., “I don’t find it easy to take charge of a situation,” reverse scored); (3) *openness to experience* (e.g., *“*I enjoy trying new and foreign foods”); (4) *agreeableness* (e.g., “Most people I know like me”)*;* and (5) *conscientiousness* (e.g., “I keep my belongings neat and clean”). Each factor comprises 12 items with a response scale from 1 (strongly disagree) to 5 (strongly agree). To assess individual differences in general emotion regulation strategies (suppression and reappraisal), we obtained self-ratings of emotion experience and expression using the emotion regulation questionnaire (ERQ) (Gross and John, 2003; Abler and Kessler, 2009), the Emotion Regulation Skills Questionnaire (ERSQ) (Berking and Znoj, 2008), and the Emotional Competence Questionnaire (ECQ) (Rindermann, 2009). The ERQ (Gross and John, 2003) (German version by Abler and Kessler, 2009) consists of 10 items and includes two subscales: (1) reappraisal based on six items (e.g., “I control my emotions by changing the way I think about the situation I am in”) (*Reappraisal*) and (2) suppression based on four items (e.g., “I control my emotions by not expressing them”) (*Suppression*). The items are rated on a scale from 1 (strongly disagree) to7 (strongly agree). The ERSQ comprises 27 items and includes 9 subscales: (1) attention, (2) body perception, (3) clearness, (4) understanding, (5) regulation, (6) acceptance, (7) tolerance, (8) self-support, (9) confrontation. The items are rated on a scale from 0 (not at all) to 4 (nearly always). The ECQ examines four competencies: (1) perception of own feelings, (2) perception of feelings of others, (3) regulation of own feelings, (3) emotional expression with 47 items. The items are rated on a five point scale from 1 (strongly disagree) to 5 (strongly agree). Alexithymia, the inability to describe and regulate one’s emotions, was assessed using the TAS-20 (Bach et al., 1996). The TAS-20 consists of 20 items and is based on a three-factor structure: (1) difficulty identifying feelings (*TAS-DIF*) (e.g., “I am often confused about what emotion I am feeling”); (2) difficulty describing feelings (*TAS-DDF*) (e.g., “I find it hard to describe how I feel about people”); and (3) externally oriented thinking (*TAS-EOT*) (e.g., “I prefer to just let things happen rather than to understand why they turned out that way”). Items are rated on a five-point Likert scale from 1 (completely disagree) to 5 (completely agree). In addition, subjects rated their ability to regulate their emotions in all experimental conditions in a separate general questionnaire at the end of the experiment on a scale from 1–100% (1: “not successful at all” to 100: “very successful”). In two open questions, subjects were given the opportunity to provide additional comments on their emotion regulation strategies (1) “Which strategy did you use to regulate your emotions? Please describe it in a few words.” The results of these questionnaires are summarized in **Table 2**. Our sample did not differ from population norms for these questionnaires (Laux et al., 1981; Bach et al., 1996; Borkenau and Ostendorf, 1999; Abler and Kessler, 2009; Rindermann, 2009; Berking et al., 2010). All questionnaires were presented in German.

**Table 2:** Results of personality questionnaires.

	*M*	*SD*
STAI pre	47.45	7.96
STAI post	57.63	8.28
Difficulty Identifying Feelings (TAS)	13.09	2.13
Difficulty Describing Feelings (TAS)	16.59	3.55
Externally Oriented Thinking (TAS)	24.59	3.50
Total TAS Score	54.27	7.44
Neuroticism (NEO-FFI)	3.10	0.64
Extraversion (NEO-FFI)	3.26	0.65
Oppenness (NEO-FFI)	4.01	0.49
Agreeableness (NEO-FFI)	3.62	0.48
Conscietiousness (NEO-FFI)	3.40	0.68
Identifying own emotions (ECQ)	2.88	0.68
Identifying others’ emotions (ECQ)	3.71	1.04
Regulation and control of own emotions (ECQ)	3.09	0.81
Emotional expressivity (ECQ)	2.86	0.68
Emotion Regulation Skills Questionnaire (ERSQ)	101.04	14.99
Reappraisal (ERQ)	2.75	1.33
Suppression (ERQ)	4.22	1.51

### Experimental Design and Procedure

**FIGURE 1:**
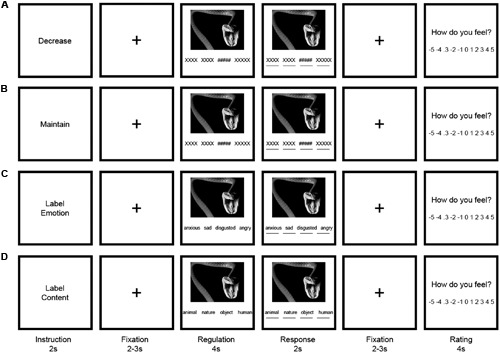
**Trial design for (A)** Decrease, **(B)** Maintain, **(C)** Label Emotion, and **(D)** Label Content conditions. Only the English version of the task is illustrated.

Our task adapted one explicit and two implicit emotion regulation conditions (**Figure 1**). In the *Decrease* condition (Reappraisal, **Figure 1A**) subjects were asked to reduce the intensity of the negative emotion by distancing themselves from the image (reappraise via perspective taking; Webb et al., 2012). This means participants should try to alter the impact of the emotional stimulus by adopting a more or less objective perspective. Before the beginning of the experiment they were proposed strategies to achieve this, such as becoming a detached observer. Importantly, participants were told not to substitute negative emotions with positive emotions. In the *Maintain* condition (control condition, **Figure 1B**), subjects viewed pictures and were asked to respond naturally to the emotional stimulus, i.e., participants should let their feelings flow without trying to regulate them (experience naturally; Webb et al., 2012). In the *Label Emotion* condition, participants were instructed to select one word that best described the evoked emotion from four emotion words presented below the picture (anxious, sad, disgusted, angry, **Figure 1C**). In the *Label Content* condition, participants were asked to select one out of four nouns presented below the picture that was most applicable to the content of the picture (human, animal, nature, object, **Figure 1D**).

Each trial started with an instruction screen for 2 s, cueing one of the four experimental conditions. After a fixation phase (2–3 s) a picture was presented for 4 s, during which participants were asked to behave according to the instructions. After this regulation phase, participants selected an appropriate response within 2 s. To keep the motor response the same for all conditions, participants pressed the button that corresponded to the hash keys in the “maintain” and “decrease” conditions. Participants’ reaction times (RTs) as well as responses were recorded for analyses. An emotional state rating followed after a short fixation phase (2 s). Participants were asked to rate their current emotional state on a scale from -5 (very negative) to +5 (very positive) within 4 s, providing a measure of trial-by-trial emotion regulation success.

Participants performed 10 experimental blocks of alternating language contexts, with the language of the first block counter-balanced across participants. Each block contained 20 trials (5 trials/condition) in which all written instructions and labels were provided in the same language, to ensure a consistent language context. Before the main experiment, participants practiced the different experimental conditions in 16 training trials.

### Stimulus Material and Online Pre-study

The stimulus set consisted of 137 pictures from the International Affective Picture System (IAPS) (Bradley and Lang, 2007) and 63 pictures from the Nencki Affective Picture System (NAPS) (Marchewka et al., 2014), which were selected from a larger pool of 300 aversive pictures based on a pilot online rating study with 145 participants. For the pilot study, 300 aversive images that could be clearly assigned to at most two of four content labels (human, animal, object, nature) were selected from the IAPS and NAPS databases. Each participant of the pilot study saw a subset of 30 pseudo-randomly chosen pictures and was asked to label the emotion that it evoked out of five choice alternatives: sad, angry, disgusted, anxious, or other. For the main experiment, 200 pictures with most unambiguous emotion labels were chosen based on the following criteria: (1) the most frequent emotion label was not ‘other’; (2) The difference in % choices between the two most frequently chosen emotion ratings was above 10%. These 200 images were distributed across eight stimulus lists matched on mean valence and arousal values, the number of images in each emotion label category, and the number of images in each content label category (**Table 3**). Moreover, images with similar semantic contents (i.e., injured babies, car crashes, assaults) were distributed evenly across stimulus lists. The eight lists were pseudo-randomly assigned to one of the six experimental conditions for each participant, whereby two lists were assigned to the ‘maintain’ condition in each language. Each stimulus list was further divided into five subsets matched on valence, arousal, and distribution of content and emotion labels, which were then assigned to blocks. Thus single blocks of the experiment were matched for these variables.

**Table 3:** Properties of stimulus pictures in each of the eight stimulus lists.

	Valence^1^		Arousal^2^		Number of items per content label			
	Mean	*SD*	Mean	*SD*	Human	Nature	Object	Animal
List 1	2.57	0.46	5.07	0.93	17	1	3	4
List 2	2.42	0.53	5.6	0.86	17	1	3	4
List 3	2.61	0.67	5.14	0.94	17	1	3	4
List 4	2.73	0.68	5.24	0.98	16	1	4	4
List 5	2.55	0.52	5.33	0.89	16	1	4	4
List 6	2.59	0.58	5.3	1.13	17	1	3	4
List 7	2.71	0.63	5.68	0.97	16	1	2	6
List 8	2.7	0.65	5.33	0.97	15	1	3	6

*^1^Based on a 9-point scale, with 1 indicating negative valence and 9 indicating positive valence.*

*^2^Based on a 9-point scale, with 1 low arousal and 9 indicating high arousal.*

During the experiment images were presented in the middle of the screen against a black background with an 800 × 600 pixel display using the stimulation software Presentation (Version 14.1, Neurobehavioral Systems, USA).

### Data Analyses

First, we identified participants that successfully regulated their emotions in their native language German. Emotion regulation success was defined as the mean decrease in self-reported negative affect when applying a cognitive reappraisal strategy to the images (*Decrease*) relative to the mean affect ratings of the control condition (*Maintain*), the latter representing the unregulated emotional response to the stimuli. Only data from effective regulators (*n* = 22) were analyzed in all further analyses. This analysis ensured that any effects in the present bilingual setting are due to differences between languages and not to the participants’ emotion regulation ability *per se*.

Data were analyzed in repeated-measures within-subject analyses of variance (ANOVA). For the follow-up planned comparisons Bonferroni-corrections were applied if necessary, indicated as *p*_B_. We report effect sizes as the generalized eta-squared ($\eta_{G}^{2}$, Bakeman, 2005) for significant effects. Where appropriate, *p*-values were corrected for violations of the sphericity assumption.

## Results

### Emotion Regulation Success

There was no difference in subjective self-reported emotion regulation success between L1 and L2, *t*(21) = 1.49, *p* = 0.15.

### Affect Ratings Following Emotion Regulation in L1 and L2

**FIGURE 2:**
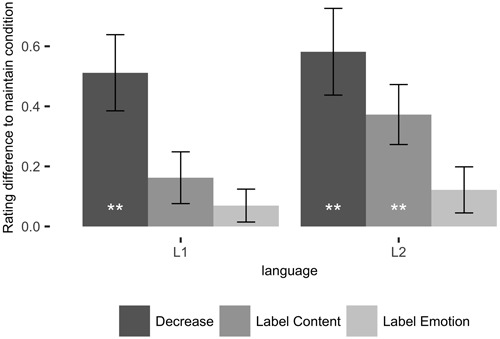
**Mean difference in affect ratings between emotion regulation conditions and maintain condition in first (L1) and second (L2) language.** Error bars indicate standard error for the comparison to the maintain condition of the respective language. **p < 0.01.

Mean affects ratings are presented in **Figure 2** and **Table 4**. The repeated-measures ANOVA of participants’ affect ratings with within-subject factors language and condition revealed a significant main effect of condition, *F*(3,63) = 10.7, *p* < 0.001, $\eta_{G}^{2}$ = 0.047. This was due to the decrease condition being the most effective emotion regulation strategy [Decrease-Look: *t*(21) = 4.29, *p*_B_ = 0.001], followed by content labeling [Label Content-Look: *t*(21) = 3.12, *p*_B_ = 0.03, Decrease-Label Content: *t*(21) = 3.45, *p*_B_ = 0.014], while affect labeling did not reduce the evoked emotions [Label Emotion-Look: *t*(21) = 1.74, *p*_B_ = 0.6] and was significantly less effective than the other two regulation conditions [Label Emotion-Label Content: *t*(21) = 2.83, *p*_B_ = 0.06, Label Emotion–Decrease: *t*(21) = 4.04, *p*_B_ = 0.003]. The main effect of language was not significant, *F*(1,21) = 1.46, *p* = 0.2, but its interaction effect with condition was, *F*(3,63) = 3.47, *p* = 0.02, $\eta_{G}^{2}$ = 0.002. To elucidate these effects we subjected the data to three planned 2 × 2 ANOVAs, each of which included the factors language and condition with the *Maintain* condition and one of the three regulation conditions. The ANOVA with the *Maintain* and *Decrease* conditions revealed a significant main effect of condition only, *F*(1,21) = 18.6, *p* < 0.001, $\eta_{G}^{2}$ = 0.08, which was due to more positive affect ratings in the *Decrease* than in the *Look* condition in both languages. This effect did not differ between languages, *F*(1,21) = 0.79, *p* = 0.38. The ANOVA with the *Maintain* and *Label Content* conditions revealed a main effect of condition, *F*(1,21) = 9.88, *p* = 0.004, $\eta_{G}^{2}$ = 0.02, due to overall more positive ratings in the *Label Content* than in the *Maintain* condition. Moreover, a significant interaction effect of condition and language, *F*(1,21) = 10.41, *p* = 0.004, $\eta_{G}^{2}$ = 0.003, was due to a stronger regulatory effect during *Label Content* in participants’ L2 (English) than in their L1 (German). Indeed, the difference between the *Label Content* and *Maintain* conditions was significant in participants’ L2, but not in their L1 [L1: *t*(21) = 1.92, *p*_B_ = 0.12, L2: *t*(21) = 3.82, *p*_B_ = 0.002]. Finally, the ANOVA with the *Look* and *Label Emotion* conditions revealed no significant effects [main effect of language *F*(1,21) = 0.04, *p* = 0.82, main effect of condition *F*(1,21) = 2.97, *p* = 0.09, interaction effect *F*(1,21) = 0.59, *p* = 0.45], which was due to a lack of regulatory effects in emotion labeling^1^.

**Table 4:** Mean affect ratings and labeling reaction times during the emotion regulation task.

Language block	Condition	Affect rating		Reaction times [ms]	
		Mean	*SD*	Mean	*SD*
L1 (German)	Look	-1.60	1.52	605	350
	Decrease	-1.08	1.65	629	396
	Label Emotion	-1.53	1.55	659	489
	Label Content	-1.44	1.73	609	393
L2 (English)	Look	-1.61	1.54	625	404
	Decrease	-1.03	1.58	652	401
	Label Emotion	-1.49	1.48	691	501
	Label Content	-1.24	1.68	608	402

### Accuracy and Reaction Times in Emotion and Content Labeling Conditions

Participants’ accuracy in the content labeling task ensured that they had understood the task correctly, as they only made 2% errors on average in both languages. Accuracy could not be defined for the emotion labeling condition, as it required a subjective emotional assessment. RTs were subjected to a 2 (language) × 4 (condition) repeated-measures ANOVA to investigate whether the four conditions differed in complexity. Although numerically RTs were slightly higher in the *Label Emotion* condition (**Table 4**), the main effect of condition was not significant (*p* = 0.09).

### Effect of Arousal on Emotion Regulation

**FIGURE 3:**
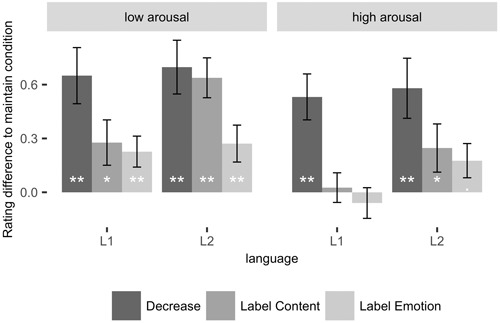
**Magnitude of emotion regulation success through reappraisal (Decrease condition), distraction (Label content), and emotion labeling, in comparison to the look condition in the respective languages.** High values indicate more effective emotion regulation. Error bars represent standard errors for the simple effects of emotion regulation success in each condition. **p < 0.01, *p < 0.05, ^∙^*p* < 0.1.

Our results showed a surprising lack of regulation by emotion labeling, challenging previous findings (Lieberman et al., 2011). However, Lieberman et al. (2011) showed that the success of emotion labeling is reduced for more arousing materials. We thus hypothesized that the lack of effect in our data might stem from the high arousal values in our stimuli. To test this, we used a median split to separate our image material into high (mean arousal: 6.1; mean valence: 2.4) and low (mean arousal: 4.5; mean valence: 2.8) arousal images. We then repeated the above ANOVA with the additional factor arousal (**Figure 3** and **Table 5**). In accord with our previous analyses, we found a significant main effect of condition, and a significant interaction of condition and language. Additionally, the main effect of arousal was due to overall more negative ratings for more arousing images. Notably, the interaction of condition and arousal was also significant. To elucidate this effect, we analyzed the interaction contrast in ANOVAs comparing the *Maintain* condition to each of the three emotion regulation conditions separately. Arousal had no effect on the difference between the *Maintain* and the *Decrease* condition, *F*(1,21) = 2.32, *p* = 0.1. However, *Content Labeling* attenuated negative affect for low arousing but not for high arousing pictures, interaction *F*(1,21) = 15.5, *p* < 0.001, $\eta_{G}^{2}$ = .007, low arousing images *t*(21) = 4.4, *p*_B_ < 0.001, high arousing images *t*(21) = 1.53, *p* = .14. Finally, the difference between the *Label Emotion* and the *Maintain* condition was also smaller for high than for low arousing images, *F*(1, 21) = 8.6, *p* = 0.007, $\eta_{G}^{2}$ = 0.003. In this analysis *Label Emotion* also significantly reduced the negative affect induced by the negative images, *F*(1,21) = 6.53, *p* = 0.02, $\eta_{G}^{2}$ = 0.007. However, this effect was due to a significant difference between the conditions for low arousing images only, *F*(1,21) = 13.26, *p* = 0.002, $\eta_{G}^{2}$ = 0.02, but not for high arousing images, *F*(1,21) = 0.43, *p* = 0.5. In summary, this analysis showed that the success of emotion regulation strategies crucially depends on the intensity of the arousing materials, but that this effect is not modulated by language context.

**Table 5:** Results of ANOVA including image arousal as additional factor.

Effect	*F*	*p*-value	Sign.	$\eta_{G}^{2}$
Condition	16.67	<0.001	*	0.052
Language	2.17	0.156		0.002
Arousal	140.38	<0.001	*	0.076
Condition : language	6.78	<0.001	*	0.003
Condition : arousal	4.96	0.004	*	0.003
Language : arousal	0.02	0.883		0.000
Condition : language : arousal	0.91	0.441		0.001

## Discussion

The present study investigated how second language context alters the processing of emotional pictures and subsequent emotion regulation via explicit and implicit strategies. We compared emotional responses evoked by negative pictures in the absence of emotion regulation (*maintain condition*) to emotional responses following explicit emotion regulation through reappraisal (*decrease condition*), as well as implicit emotion regulation through *emotion labeling* and *content labeling* in each of our bilingual participants’ two languages.

### Emotion Regulation in First and Second Language

*Content labeling* regulated emotions better in the L2 than in the L1 indicating a previously not reported L2 advantage in implicit emotion regulation through distraction. This finding supports the notion of decreased automaticity of affective processing in L2, as previously found for linguistic stimuli (Pavlenko, 2012; Caldwell-Harris, 2015). Labels in the foreign language might be more distracting than labels in the native language, capturing more attention. In this view, distraction relies on attentional control to focus on a concurrent task (content labeling) that is unrelated to the emotion, thereby reducing emotional responding and establishing more distance to the emotional stimuli and one’s own emotional response (Webb et al., 2012). As distraction occurs early in the emotion regulative process (Gross, 2002), foreign language use might successfully help to modulate emotions as soon as they arise and thus shorten the duration and decrease the magnitude of emotional responses. Thus, future studies should implement objective physiological measures of emotional responses such as skin conductance or EEG responses to provide further insight into the temporal dynamics underlying the second language effect in emotion regulation.

Importantly, we find this effect in a consistent L2 setting, supporting the notion that emotional distancing through foreign language use is not confined to situations that require frequent language switching, at least not for highly emotional tasks such as emotion regulation (Oganian et al., 2015). Since the same labels were used throughout the task and participants’ familiarity with the labels was ensured at the beginning of the experiment this is also unlikely an effect of novelty of the labels. In contrast, language context had no effect on *emotion labeling*. One possible reason for this is that emotion labeling requires direct assessment of evoked emotions, counteracting the distance induced by the L2 context. Future studies should test whether this pattern might differ in a more speeded task setting.

Finally, the effects of *cognitive reappraisal* did not differ between language contexts. However, as we did not monitor the language of potential inner speech during cognitive reappraisal it is possible that our study participants relied on their native language in this condition, thus acting against the foreign language context. Furthermore, in absence of emotion regulation (*maintain condition*) we found no differences in affect induced by negative pictures that were embedded in bilinguals’ L1 and L2. These findings suggest that incidental foreign language context *per se* is not sufficient to reduce induced affect.

Reduced affect in second language settings has also been discussed in the framework of embodiment theories (Foroni and Semin, 2009). It has been argued that the use of a foreign language acquired in contextually less rich classroom settings is less embodied (i.e., not or to a lesser extend inducing simulations of perceived content), resulting in less affect (e.g., Foroni, 2015). Although our experimental design allows no insight into theories of embodiment, we want to note that increased emotion regulation is compatible with findings of reduced motor co-activation for negative stimuli in a foreign language. Future studies are needed to investigate the link between embodiment, emotion regulation and foreign language.

### Implicit and Explicit Emotion Regulation

In participants’ native language context (German), our results corroborate and extend previous studies of emotion regulation by providing a direct comparison between three different emotion regulation strategies under conditions of high and low arousal. *Reappraisal* proved to be a successful emotion regulation strategy for high and low arousal images alike. It was also more effective than *emotion* and *content labeling* in reducing self-reported feelings of unpleasantness (Lieberman et al., 2011; Webb et al., 2012; Burklund et al., 2014). Furthermore, *content labeling* was more effective than *emotion labeling* (Constantinou et al., 2015), and both implicit emotion regulation strategies were more successful for low-arousing images. While *content labeling* had some effect even for high-arousing images, *emotion labeling* down-regulated induced affect for low-arousing images only (Lieberman et al., 2011).

It has to be noted, that *emotion labeling* does not always imply the down-regulation of emotional responses (Lieberman et al., 2011). Putting feelings into words includes a number of sub-processes that might not be beneficial to effective emotion regulation (Moyal et al., 2014): First, attention is directed to the affective features of a stimulus resulting in higher engagement with the emotional response. Second, labeling requires categorizing feelings and separates the feelings from one. This process could be difficult for the highly complex IAPS pictures eliciting a mixture of emotions. The hindered emotion categorization might result in less effective emotion regulation.

The different effects of arousal on explicit and implicit emotion regulation add another dimension to current process models of emotion regulation. So far, emotion regulation has been viewed along a timescale, categorizing different strategies from early (e.g., distraction) to late (e.g., suppression) along the emotion regulative process involving different cognitive sub-processes (e.g., attention, appraisal, response inhibition) (Gross, 2002; Koole, 2009). Our data demonstrate that arousal might affect early emotion regulative processes such as attention, whereas later components such as appraisal might be less affected. Thus, the modulatory role of arousal should to be considered in future studies that focus on early emotion regulation processes.

The effect of language use on emotional processing and regulation ability is also of interest to real-world clinical contexts. A recent meta-analysis showed that psychotherapy in the native language is on average twice as effective as therapy in a foreign language (Griner and Smith, 2006). Furthermore, clinical case studies of bilinguals in therapy and psychoanalysis provide evidence for differences in language emotionality and indirectly relate to emotion regulation (e.g., Buxbaum, 1949; Aragno and Schlachet, 1996; Movahedi, 1996; Schwanberg, 2010). Suppressing emotions was effective in L2 but not in L1, e.g., a single word in L1 was sufficient to evoke childhood memories and feelings of anxiety and fearfulness (Aragno and Schlachet, 1996). In line with this, our results demonstrate that regulatory processes can benefit from foreign language use. It has also been shown that L2 functions as an asylum for patients, enabling them to feel safe and distant when discussing highly emotional and/or traumatic experiences (Buxbaum, 1949; Movahedi, 1996). As our present study explores a setting where negative affect and regulation happened in the same language context, further studies should address the cognitive mechanisms underlying such effects of incongruence between stimulation language and evaluation or regulation languages. Furthermore, it would be important to delineate the effects of foreign language proficiency and frequency of use on emotional regulation a foreign language context.

## Conclusion

Our findings indicate that foreign language context *per se* does not alter emotional experiences of non-linguistic stimuli, but that foreign language use is advantageous over the native language for certain emotion regulation techniques.

## Author Contributions

CM and YO designed the experiment, collected data, analyzed data, and wrote the manuscript. US helped collecting data. AJ and HH helped design the experiment and write the manuscript.

## Conflict of Interest Statement

The authors declare that the research was conducted in the absence of any commercial or financial relationships that could be construed as a potential conflict of interest.

## Footnotes

1 A way to further understand the effects of L2 on emotion regulation would be to look at the correlations between participants’ L2 proficiency and emotion regulation success in the L2. While we did not find a significant relationship between proficiency and various measures of regulation success in our data, we are wary of over-interpreting this result, due to the small size of our sample and the homogeneous L2 proficiency levels of our participants.
